# An Interesting Case of Isolated Cysticercosis of Breast Masquerading as a Breast Lump in a Young Female

**DOI:** 10.7759/cureus.46550

**Published:** 2023-10-05

**Authors:** Aditya Sharma, Mumtaz A Ansari, Arvind Pratap, Vivek Srivastava

**Affiliations:** 1 Department of General Surgery, Institute of Medical Sciences, Banaras Hindu University, Varanasi, IND

**Keywords:** parasitic infections, fine needle aspiration cytology (fnac), breast cysticercosis, breast lump, human cysticercosis

## Abstract

Human cysticercosis can affect any tissue or organ in the body and may be asymptomatic or manifest clinical signs and symptoms depending on the area of the body where cysticerci are found. However, at the same time, the involvement of the breast by cysticercosis is an extremely rare phenomenon, with very few case reports published before. In this report, we present the case of a 26-year-old married woman who came with a history of painless swelling in the left breast.

## Introduction

Human cysticercosis is a serious public health issue, particularly in developing nations, caused by *Cysticercus cellulosae *[1]. Skeletal muscle, subcutaneous tissue, the breast, the brain, and the eye are the most often affected organs by cysticercosis, in decreasing order of frequency [2]. Cysticercosis can occur in the breast; however, it is not uncommon. In the existing literature, very few cases have been mentioned [3]. Patients frequently exhibit symptoms in the form of breast lumps or cysts [4]. In this case report, we discuss a rare case of cysticercosis in the breast that manifested as a small, non-tender lump.

## Case presentation

**Figure 1:**
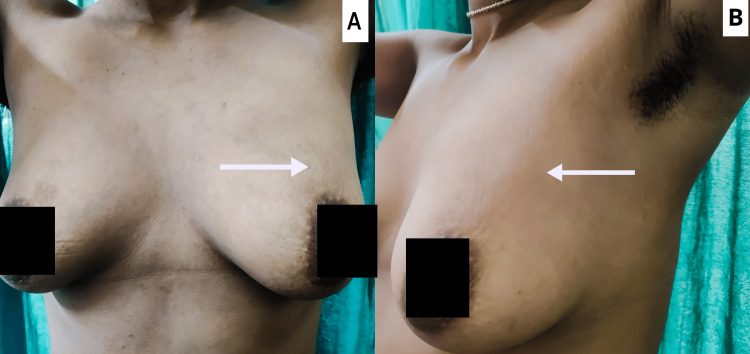
(A) A clinical image showing the exact location of the left breast lump in the upper outer quadrant (marked by an arrow). (B) An oblique view of the swelling.

A 26-year-old female vegetarian belonging to a middle-class family presented to the surgery outpatient department with chief complaints of swelling in her left breast in the upper quadrant for the past four months. There were no associated comorbidities, and there was no history of animal contact in the family. On examination, a lump of size 2.5x2.5 cm in the upper outer quadrant of the left breast, with a hard, irregular surface and firm consistency and free from the overlying skin but fixed to the underlying muscle, was observed, as shown in Figure 1.

**Figure 2:**
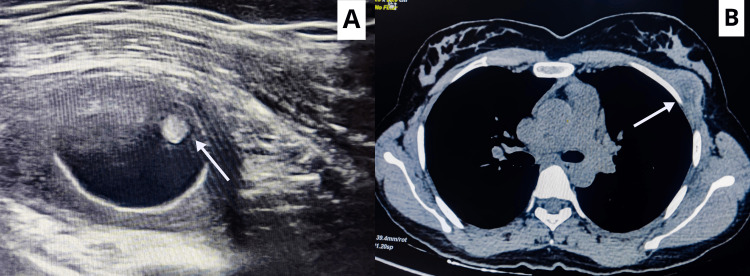
(A) Ultrasonography of the left breast reveals a well-defined oval cyst within a collection and a brightly echogenic protrusion from the wall. (B) A contrast-enhanced computed tomography film showing a cystic lesion in the left anterolateral chest wall in the intermuscular plane of the pectoralis minor muscle.

The examination of bilateral axillae was within normal limits. The hematological parameters, such as eosinophils and erythrocyte sedimentation rate, were within normal limits. Ultrasonography of the left breast revealed a well-defined oval cyst within a collection and a brightly echogenic protrusion from the wall. The contrast-enhanced computed tomography (CECT) of the thorax revealed a well-defined, heterogeneously enhancing hypodense soft tissue density lesion of approximately 2.3x2.22 cm with internal hyperdense foci noted in the left anterolateral chest wall in the intermuscular plane of the pectoralis minor muscle features suggestive of intermuscular cysticercosis, as shown in Figure 2.

Fine needle aspiration cytology (FNAC) revealed a clear fluid, and the cytology of the fluid showed eosinophils and macrophages. There were no parasites isolated from the stool examination. The CECT of her brain and ophthalmic examination were within normal limits. Based on these findings and with the patient belonging to the endemic region, a diagnosis of parasitic infestation was made. A biopsy from the swelling was not considered in this patient. The key to establishing the probable preoperative diagnosis of a parasitic breast disease was this high index of suspicion and awareness, considering the endemic region she was coming from.

She was started on albendazole oral administration at a dose of 15 mg/kg/day in two daily doses for four weeks, and she responded well to the medical management. In subsequent visits, the cyst was completely resolved on radiological imaging, and no surgical intervention was required.

## Discussion

Human cysticercosis happens when eggs of the parasite are ingested or when an adult worm regurgitates ova into a human stomach [5]. The larvae are released in the stomach, pass through the intestinal mucosa, and are then transported to various areas of the body where they develop cysticerci, a 0.5-1 cm cyst that houses the head of the juvenile worm. They neither expand their size nor move [6]. Skeletal muscles, subcutaneous tissues, the brain, and the eye are common locations [7].

Cysticercosis in the breast is still uncommon. Only eight incidences of cysticercosis were found in 8,364 breast aspirates at an apex Indian medical institution. A total of 28 cases of breast parasites (16 cases of cysticercus and 12 cases of filariasis), identified by FNAC during a 21-year period, were examined to determine the manner in which the host tissue responded [8]. Out of 23,402 biopsy results obtained from a study conducted in Nepal, 62 cases of cysticercosis were found, with 8% of those cases being found in the breast [9].

The breast is a rare location. The majority of cases of taeniasis and cysticercosis are discovered in rural, undeveloped areas of developing nations with subpar sanitation [10]. Such lesions could be easily appreciated on ultrasonography or CECT and be managed either by giving a medical trial of a benzimidazole anthelmintic, such as albendazole, as in our case, or surgically if the lesion is not responding to these drugs.

## Conclusions

Cysticercosis of the breast is a rare entity, with only a few cases reported before, and should be considered a differential diagnosis for a lump in the breast, especially in the endemic regions. It might resemble a breast tumor clinically. The diagnostic utility of FNAC has increased, attributable to the cytomorphological identification of larvae in smears. When clear fluid is aspirated, suspicion regarding the parasitic lesion immediately arises due to the presence of eosinophils, neutrophils, palisading histiocytes, and giant cells. For establishing a preoperative diagnosis, results from imaging are helpful, as seen in our case.
